# Prenatal maternal mental health and neurodevelopment in congenital heart disease in France: the neuro-moms CHD multicentre prospective study protocol

**DOI:** 10.1136/bmjopen-2025-104889

**Published:** 2025-12-23

**Authors:** Julie Deninotti, Neil Derridj, Sylvie Martins, Daniela Laux, Bertrand Stos, Marilyne Levy, Beatrice Desnous, Sophie Guillaumont, Pascal Amédro, Julie Chabaneix, Marion Pfister, Gaelle Marguin, Gwladys Desmure, Marie Vincenti, Damien Bonnet, Johanna Calderon

**Affiliations:** 1 U1046 PhyMedExp, National Institute of Health and Medical Research, Montpellier, France; 2 M3C-Necker, Hôpital Universitaire Necker-Enfants Malades, AP-HP, Université de Paris Cité, Paris, France; 3 Timone Children Hospital, Marseille, France; 4 University Hospital of Montpellier, Montpellier, France; 5 University Hospital Centre Bordeaux Cardiology Hospital, Pessac, France; 6 Patient Association Petit Coeur de Beurre, Paris, France; 7 UMR 1163 Imagine Institute, University Paris Cité, INSERM, Paris, Île-de-France, France

**Keywords:** Developmental neurology & neurodisability, Congenital heart disease, Psychological Stress

## Abstract

**Abstract:**

**Introduction:**

Neurodevelopmental impairments in congenital heart disease (CHD) are the most frequent long-term morbidity. Adverse neurodevelopmental outcomes may start in the prenatal period. Maternal mental health may be a potentially modifiable risk factor for the optimisation of neurodevelopment in CHD. We propose to assess the impact of prenatal maternal mental health on 1-year neurodevelopmental outcomes in complex CHD.

**Methods and analysis:**

Neuro-Moms CHD is a national multi-centre, prospective study of prenatal maternal mental health and neurodevelopmental outcomes in children with complex CHD who undergo neonatal open-heart surgery. Participants (n=87 mother-child dyads) will be recruited from five major French paediatric cardiology centres (Necker Children’s Hospital in Paris, Bordeaux Cardiology Hospital, Marseille Children’s Hospital, Montpellier University Hospital and Saint-Pierre Institute). Expecting women who receive a prenatal diagnosis of fetal complex cyanotic CHD that requires a neonatal open-heart surgery for the newborn are eligible to participate. They will complete self-reports on mental health, anxiety, depression and coping skills and will participate in a semi-structured psychological interview. Mothers will provide information on medical, sociodemographic and lifestyle factors. They will be enrolled during the third trimester of pregnancy and will participate at three time points: prenatal, T1; after the newborn’s cardiac surgery, T2; and between 12 and 18 months after birth of the child with CHD, T3. Children with CHD will undergo a standardised neurodevelopmental assessment when they turn 12–18 months old. The father or co-parent of the child with CHD will also participate in T1 and will complete mental health self-reports. We will use a structural equation model to estimate simultaneously the relationships among maternal mental health, prenatal factors and child neurodevelopment outcomes.

**Ethics and dissemination:**

This study is sponsored by the French National Institute of Health and Medical Research. It was approved by the Ethics Committee on 5 November 2024 and is registered in a public trials registry (NCT06711666). Neuro-Moms CHD targets a public health question with important societal implications. Results are expected to be broadly communicated with the scientific community and the lay public. Dissemination of findings will be in the form of scientific articles in peer-reviewed journals and presentations at conferences. Any publication or communication will comply with the international recommendations: ‘Recommendations for the Conduct, Reporting, Editing, and Publication of Scholarly work in Medical Journals’ (http://www.icmje.org/recommendations). All participants will give written informed consent or assent to participate. The anonymised data to be collected in this study will be available within the manuscripts published.

**Trial registration number:**

NCT06711666; pre-results.

**STRENGTHS AND LIMITATIONS OF THIS STUDY**

- This is a national, multi-centre study, which will help to improve the care pathway for children with congenital heart disease (CHD).
- In addition to the assessment of mental health in the prenatal period using standardised self-report questionnaires, a 45 min semi-directive interview will be conducted by a psychologist with expertise in perinatal mental health, enabling a more detailed assessment of the complexity of mental health in the prenatal period in expecting mothers with a CHD diagnosis.
- This study is longitudinal and will provide a neurodevelopmental assessment at age 1 for children with CHD, but neurodevelopment is a long trajectory: children’s cognitive, behavioural and socio-emotional development should be evaluated again at different ages and stages of their life.

## INTRODUCTION

 Congenital heart disease (CHD) represents 1% of live births and is the leading cause of congenital malformations at birth.^1 2^ Advances in cardiac surgery and medical management of CHD have increased the survival and life expectancy of children with the most complex forms of CHD through interventions performed during the neonatal period. This increase in the population living with cyanotic CHD has been accompanied by an increased recognition of their long-term morbidities. North American guidelines from the American Heart Association consistently highlight the fact that neurodevelopmental outcomes in CHD are a pressing public health issue.^3 4^

Neurodevelopmental impairment in children born with CHD has a multifactorial aetiology, with a probable synergy between risk factors that has yet to be elucidated.^5^ Many factors interact and/or accumulate which contribute to these long-term morbidities. It has been established that the origins of neurodevelopmental impairments may start in the prenatal period,^6^ or even at conception, with some genetic overlap between CHD and neurodevelopmental disorders.^7^ Pathophysiological mechanisms, already observed in the prenatal period, suggest that certain CHD described as cyanotic, like Transposition of the Great Arteries (TGA) or Hypoplastic Left Heart Syndrome, can influence early brain development by inducing hypoxia and/or cerebral hypoperfusion.^8 9^ As a result, the fetal brain develops in conditions of insufficient oxygen supply, leading to a dysmature brain with lesions detectable as early as the second trimester of pregnancy.^10^

The neonatal period is also crucial for children with complex CHD, marked by insults on the developing brain, such as frequent episodes of hypoxia and heart failure, which can aggravate damage to an already vulnerable brain. Postnatal diagnosis of critical CHD,^11 12^ neonatal interventions, including cardiac surgery and selective cerebral perfusion,^13^ are also factors considered in the onset of neurodevelopmental impairments. To date, distinguishing causal factors from intermediate or confounding factors remains a complex task. Initially, cardiac surgery and related surgical and medical factors were thought to be among the major risks implicated with neurodevelopmental disorders in CHD.^14^ However, despite advances in surgical techniques and management, these disorders persist, suggesting that the variance attributable to the medical management of CHD may not be dominant. Importantly, lifestyle factors, such as the child’s living environment and socioeconomic factors (economic status, accessibility to care, parental education),^15^ also appear to influence these impairments.

It is increasingly recognised that prenatal risk factors play a prominent role in the neurodevelopmental prognosis for children with critical CHD. Recent data obtained from a cohort of pregnant women whose fetus was diagnosed with complex CHD showed that cerebral growth delay (ie, reduced cortical volumes) was observable in utero, and that this delay was predictive of poorer cognitive and motor prognosis at age 2.^16^

Some prenatal factors have been recognised as playing a crucial role in the emergence and severity of neurodevelopmental impairments in children with CHD.^12 17 18^ Our previous study evaluated the impact of prenatal diagnosis of one of the most studied complex cardiac malformations, TGA, on neurocognitive outcomes. Findings from this study showed that a prenatal diagnosis of TGA was associated with better neurocognitive scores, particularly in executive functions, in preschool and school-aged children.^12^ In addition, findings from the largest French population-based cohort, Epidemiology of Congenital Heart Defects (EPICARD), demonstrated that perinatal factors associated with in-utero developmental delay, such as low birth weight for gestational age, were deleterious to neurodevelopmental prognosis in CHD.^19^ In utero developmental delay has been linked to poor maternal mental health in the general population.^20^ It is unknown whether prenatal maternal psychological distress has an impact on neurodevelopmental outcomes in children with CHD.

### Maternal mental health disorders and neurodevelopmental outcomes

Maternal mental health disorders are a significant concern in public health. Pregnancy and the post-partum period, which are particularly vulnerable in terms of biological and psychological changes, significantly increase the risk of developing a mental health disorder. Impaired maternal mental health is now recognised as one of the main peripartum and post-partum morbidities, with postpartum depression affecting up to 22% of women during the prenatal period or the first post-partum year.^20^ This percentage rises in the case of complicated or high-risk pregnancies, particularly when the fetus is diagnosed with CHD.^21^,^23^ These expecting women are faced with a multitude of sources of stress, ranging from invasive complementary examinations (eg, amniocentesis) to prognostic uncertainty about the health of the unborn child. In this context, the risk of maternal mental health deterioration dramatically rises, with a particularly high incidence of depressive states, anxiety disorders and post-traumatic stress disorder (PTSD) in the perinatal period.

In the absence of a specific fetal diagnosis, maternal mental health may be linked to changes in fetal brain development in utero, as well as to potential long-term cognitive, emotional and behavioural impairment in the child.^24^,^27^ Thus, maternal mental health appears to influence the neurodevelopment of the unborn child by modulating, in utero, both cerebral maturation and postnatal psychological attachment processes.^27^ More specifically, a recent study showed that perinatal maternal depression was related to the complexity or severity of the unborn child’s CHD, and that maternal stress and anxiety were associated with smaller cerebellar and hippocampal volumes in the fetus.^28^

Maternal psychological distress may be a potentially modifiable risk factor in the optimisation of neurodevelopment in CHD. However, to date, no study has investigated the link between prenatal mental health and neurodevelopmental outcomes in children with CHD. Similarly, although studies have emphasised the importance of maternal psychological support following fetal diagnosis of CHD, very few studies have focused on understanding the determinants of maternal mental health and their potential direct or indirect impact on the neurodevelopment of children with CHD. A better understanding of these psychological, medical and sociodemographic determinants, linked to the mother and linked to the child with CHD, is essential for risk stratification in terms of both maternal mental health and the neurodevelopmental outcome of children with CHD. Understanding the impact of psychological factors on neurodevelopmental impairments in children with CHD is crucial for designing preventive interventions that enhance neurodevelopment. Neuro-Moms CHD is the first multicentre longitudinal study to focus on the impact of prenatal maternal mental health on 1-year neurodevelopmental outcomes in children with complex cyanotic CHD who undergo neonatal open-heart surgery (ie, children at high neurodevelopmental risk).

### Aims and hypotheses

#### Main objective

*Aim 1*: To characterise the impact of prenatal maternal psychological distress on neurodevelopmental outcomes at age 1 for children with cyanotic CHD who undergo neonatal open-heart surgery. We hypothesise that a higher level of maternal psychological distress as measured by the Symptom Check-List-90 R (SCL-90-R)^29^ will be associated with lower standardised neurodevelopmental scores as measured using the Bayley Scales of Infant and Young Child Development, Fourth edition (Bayley-4^30^) in children with CHD.

#### Secondary objectives

*Aim 2*: To describe the evolution of maternal mental health (ie, anxiety, depression and psychological adjustment scores), from the antenatal period to the neonatal postoperative period (after cardiac surgery), and finally when the child with CHD turns 1 year. We predict that maternal psychological distress will decrease over time, as we expect that the most acute period of stress will be closest to the initial diagnosis of fetal CHD (T1, T2 and T3).

*Aim 3*: To identify the medical and sociodemographic factors associated with prenatal maternal psychological distress of women carrying a fetus diagnosed with cyanotic CHD. We hypothesise that lower maternal educational attainment and poor family support as well as fetal factors such as diagnosis of severe CHD (ie, univentricular heart) will be associated with worse prenatal maternal mental health outcomes (ie, overall mental health, anxiety and depression symptoms). The evaluation of scores obtained on self-report questionnaires (SCL-90-R,^29^ State-Trait Anxiety Inventory (STAI-Y),^31^ Coping Inventory for Stressful Situations (CISS),^32^ Edinburgh Postnatal Depression Scale (EPDS),^33 34^ Posttraumatic Stress Disorder Checklist for DSM-5 (PCL-5)^35 36^) completed by the mother will be analysed in relation to medical, sociodemographic and lifestyle factors.

*Aim 4*: To explore the mediating role of prenatal risk factors (ie, medical factors and maternal coping strategies, such as task-oriented, avoidant and emotional coping) in the association of prenatal maternal mental health (ie, distress, anxiety and depression) and neurodevelopment in children with CHD. We will also explore the potential interaction effects between sociodemographic factors and mental health on neurodevelopment in CHD. We hypothesise that expecting mothers with low socioeconomic levels of support and/or with maladaptive coping strategies (eg, avoidant, emotional reactivity) will experience the greatest effects of prenatal distress on their children’s neurodevelopmental outcomes at 1 year of age. The mediating role of medical and psychological antenatal factors (information extracted from the medical record, the interview and the information questionnaire) on the relationship between maternal mental health scores (SCL-90-R, STAI-Y, CISS, EDPS, PCL-5) and the child’s neurodevelopment (Bayley-4,^30^ Ages and Stages Questionnaire-Third Edition (ASQ-3)^37^) will be characterised.

*Exploratory Aim 5*: To explore the impact of the mental health of the father or co-parent of the child diagnosed prenatally with CHD on the cognitive neurodevelopment score of the child at age 1. The SCL-90-R (general mental health), STAI-Y (anxiety symptoms) and CISS (coping skills) self-report questionnaires will be presented to the co-parent only in Time 1 (concomitantly with the maternal prenatal questionnaires). The Global Score Index of the SCL-90-R will be used as an indicator of the co-parent’s mental health.

## METHODS AND ANALYSIS

### Study design

This is a national multicentre, non-interventional, prospective and longitudinal study of prenatal maternal mental health and child’s neurodevelopmental outcomes in CHD (n=87 mother-child dyads). Expecting women who consent to participate will undergo psychological evaluations. These evaluations will be conducted by a psychologist with expertise in perinatal mental health. This psychological assessment is conducted at three time points:

- Time 1 (T1), during the third trimester of pregnancy (prenatal period), between 28 and 38 weeks of gestation;
- Time 2 (T2), after the child’s cardiac surgery, and before their hospital discharge (ie, in the postnatal period, after intensive care transfer to inpatient care); and
- Time 3 (T3), between 12 and 18 months after birth of the child with CHD.

The father or co-parent of the unborn child will also be invited to participate in the study at Time 1 by completing self-report questionnaires similar to those of the expecting participant. The child with CHD will be involved in a neurodevelopmental assessment when he/she is 12–18 months of age. This visit will be integrated into his/her usual cardiac neurodevelopmental care, whenever possible.

### Participants and recruitment

Participants are (1) expecting women carrying a fetus prenatally diagnosed with an isolated cyanotic CHD that is not associated with known genetic syndromes and/or major extra-cardiac anomalies; (2) their child with CHD when they turn age 12 months (+6 months maximum) at the time of the neurodevelopmental examination (T3); and the father or co-parent of the child (invited to participate during the prenatal period only). Expecting women are included if they meet the following criteria: (1) expecting woman aged ≥18 years old who received a prenatal diagnosis of isolated fetal complex cyanotic CHD confirmed by a paediatric cardiologist; (2) pregnancy at week 28 minimum (third trimester) and up to week 38 of gestation maximum; (3) a delay of 4 weeks minimum between initial fetal diagnosis of CHD and study participation; (4) women living in France and being followed-up at one of the participating cardiology centres and (5) social security in France or Europe. Children with CHD are included if they meet the following criteria: (1) received a prenatal diagnosis of isolated fetal complex CHD and child of a woman included in the study; (2) consent of both parents or legal guardians; (3) social security in France or Europe. Co-parents are included in the study if they meet the following criteria: (1) co-parent aged ≥18 years old with an expecting woman already included in the study; (2) social security affiliation in France or Europe. Only participants who were able to understand the questionnaires independently or via a direct translation, if needed (ie, only those who spoke French or English) were included in this study.

Exclusion criteria will be, for expecting women and co-parents: (1) refusal to participate; (2) fetus with CHD associated with other fetal comorbidity that has a clinical impact on neurodevelopment (ie, genetic syndromes such as trisomy 13-18-21, poly-malformative syndromes, cerebral malformations) or according to the clinical judgement of the investigator; (3) wish for medical termination of pregnancy at the time of inclusion; (4) inability to understand and/or complete the self-report questionnaires; (5) medical diagnosis of severe psychiatric disorder (eg, psychotic disorders, major depressive disorders), with or without treatment (a referral for perinatal psychiatric consultation will be proposed to patients excluded due to these conditions); (6) under court protection, guardianship or conservatorship. Exclusion criteria will be, for children: (1) genetic abnormality, cerebral malformation making it impossible to evaluate the child using the standard neurodevelopmental tools chosen for the study.

Participants will be recruited from the two French M3C (complex congenital heart defects) reference centres, Hôpital Necker Enfants Malades and CHU de Bordeaux, as well as the centres of competence for complex congenital heart defects, CHU de Montpellier, AP/HM Hôpitaux de Marseille and Institut Saint Pierre, during a fetal cardiology follow-up consultation. The Principal Investigator (PI) of the study (JC) delegates local PIs in each centre to ensure multi-site coordination. The local PIs will ensure local regulatory compliance.

Information and inclusion of participants will be carried out by the fetal cardiologists who provide antenatal consultations in congenital cardiology, or by a qualified investigator (neuropsychologist or clinical psychologist). The study will be proposed at least 4 weeks after the initial diagnosis of CHD, to provide appropriate time for the family to process and decide on the continuation of pregnancy.

### Patient and public involvement

The research questions and outcomes of this study were developed by considering mental health challenges in the context of a prenatal diagnosis of CHD, and aiming for the optimal development of the child, which is a priority for every parent. Patient association ‘Petit Coeur de Beurre’ was involved in the identification of key mental health questions for this study. They will be involved in the dissemination of results to parents of children with CHD.

### Primary outcome measures

- The primary outcome will be the relationship between the global mental health self-report questionnaire (SCL-90-R) at the prenatal evaluation (T1)^29^ and neurodevelopmental outcomes at age 1 measured via the Bayley-4.^30^ The SCL-90-R is a 90-item self-administered questionnaire assessing the psychological distress and mental health profile of adults. Each item is rated on a five-point Likert scale, from 0 (= not at all) to 4 (= extremely), according to the distress caused by each symptom. It assesses nine primary symptom dimensions: somatisation, obsessive-compulsive, interpersonal sensitivity, depression, anxiety, hostility, phobic anxiety, paranoid ideation and psychoticism. Three global indices provide measures of overall psychological distress: the global severity index (GSI), the positive symptom total and the positive symptom distress index (PSDI). The internal consistency of the SCL-90-R is satisfactory (α=0.54 to 0.84 for the French validation in adults).^28^ The GSI will be retained as the primary indicator of mental health in this study because it is the most sensitive indicator of the subject’s psychological distress, combining information on the number of symptoms and the intensity of psychological distress. The GSI is a standardised T-score (mean 50, SD 10).
- The cognitive score of Bayley-4^30^ will be used as the primary indicator of neurodevelopment in children with CHD at age 1 in this study. The Bayley-4 is a multidomain assessment tool of development in children, supporting early detection of developmental issues by measuring five key domains: cognitive, language, gross and fine motor and social-emotional. Bayley-4 scores are standardised and reported as standard scores (mean 100, SD 15). The Bayley-4 is the most widely used cognitive tests between the ages of 18 and 40 months^38^ and has solid psychometric qualities.^39 40^

### Secondary outcome measures

Secondary outcomes will be specific anxiety symptoms (STAI-Y), coping skills (CISS questionnaire), depression (EPDS questionnaire) in relation to neurodevelopmental and behavioural scores.

- The STAI-Y^31^ is a 40-item self-report questionnaire assessing trait anxiety (20 items) and state anxiety (20 items). Each item is rated on a four-point Likert scale, from 1 (= almost never) to 4 (= almost always), with higher scores indicating greater anxiety.
- The CISS^32^ is a 48-item self-report questionnaire assessing individual coping styles in response to stress. It evaluates three primary coping dimensions: task-oriented coping, emotion-oriented coping and avoidance-oriented coping. Each item is rated on a five-point Likert scale, from 1 (= not at all) to 5 (= very much), with higher scores indicating greater use of the coping style.
- The PCL-5^35 36^ is a 20-item self-report questionnaire assessing symptoms of PTSD as defined by the DSM-5. Symptom severity is evaluated across four clusters, namely: intrusion, avoidance, negative alterations in cognition and mood and arousal and reactivity. Each symptom is rated on a five-point Likert scale, from 0 (= not at all) to 4 (= extremely), depending on its severity in the last month. A score above the cut-point score of 31–33 indicates PTSD.
  The EPDS^33 34^ is a 10-item self-report questionnaire assessing postpartum depression symptoms. Each item is rated 0 to 3 depending on the frequency of symptoms in the last week. Total scores range from 0 to 30, with scores above the cut-off point of 12.5 indicating postpartum depression.
- The ASQ-3^37^ is a parental questionnaire for children aged 1 month to 5 years 6 months that screens five developmental domains: communication, gross motor, fine motor, problem-solving skills and adaptive skills. Each domain contains six items, rated 10 (=yes), 5 (=sometimes) or 0 (= not yet) by the parent or caregiver depending on their child’s current skills. The ASQ-3 is a recommended screening tool of child development with good psychometric properties.^37 41^

### Covariate measures

Medical, sociodemographic and lifestyle factors will be investigated. The sociodemographic factors collected will be maternal educational level, marital status, support systems (close family, access to medical care, level of geographical isolation, etc.), lifestyle characteristics regarding sleep, alcohol use and smoking and physical activity during pregnancy. We will derive indices of socioeconomic vulnerability from the French National Institute of Statistics and Economic Studies and include them in exploratory socioeconomic analyses. The medical factors collected will be type of CHD, gestational age, echocardiographic characteristics of the fetus (growth, associated cardiac anomalies), timing between first CHD fetal diagnosis and psychological assessments, maternal comorbidities including gestational diabetes, hypertension, placental abnormalities and any history of psychological or neurological symptoms for the mother as well as current and past pharmacological treatments. This information will be extracted from medical records, one semi-directive interview and the information questionnaire. Information on the child will also be collected by medical records and will include prenatal (spontaneous vs assisted reproductive technology, antenatal corticoid use or other neuroprotective therapies), perinatal (c-section birth, gestational age, birth weight, Appearance, Pulse, Grimace, Activity, Respiration (APGAR) score at 5 min, cardiac arrest, any other neonatal complications) and operative variables as well as current neurological diagnoses (ie, epilepsy, stroke, other events).

### Procedures

The inclusion visit at Time 0 will take place at each participating centre during the usual follow-up fetal congenital cardiology consultation after the initial diagnosis of cyanotic CHD. The investigator will inform the participant, answering all questions concerning the aims, the nature of the constraints, the foreseeable risks and the expected benefits of the research.

**Figure 1:**
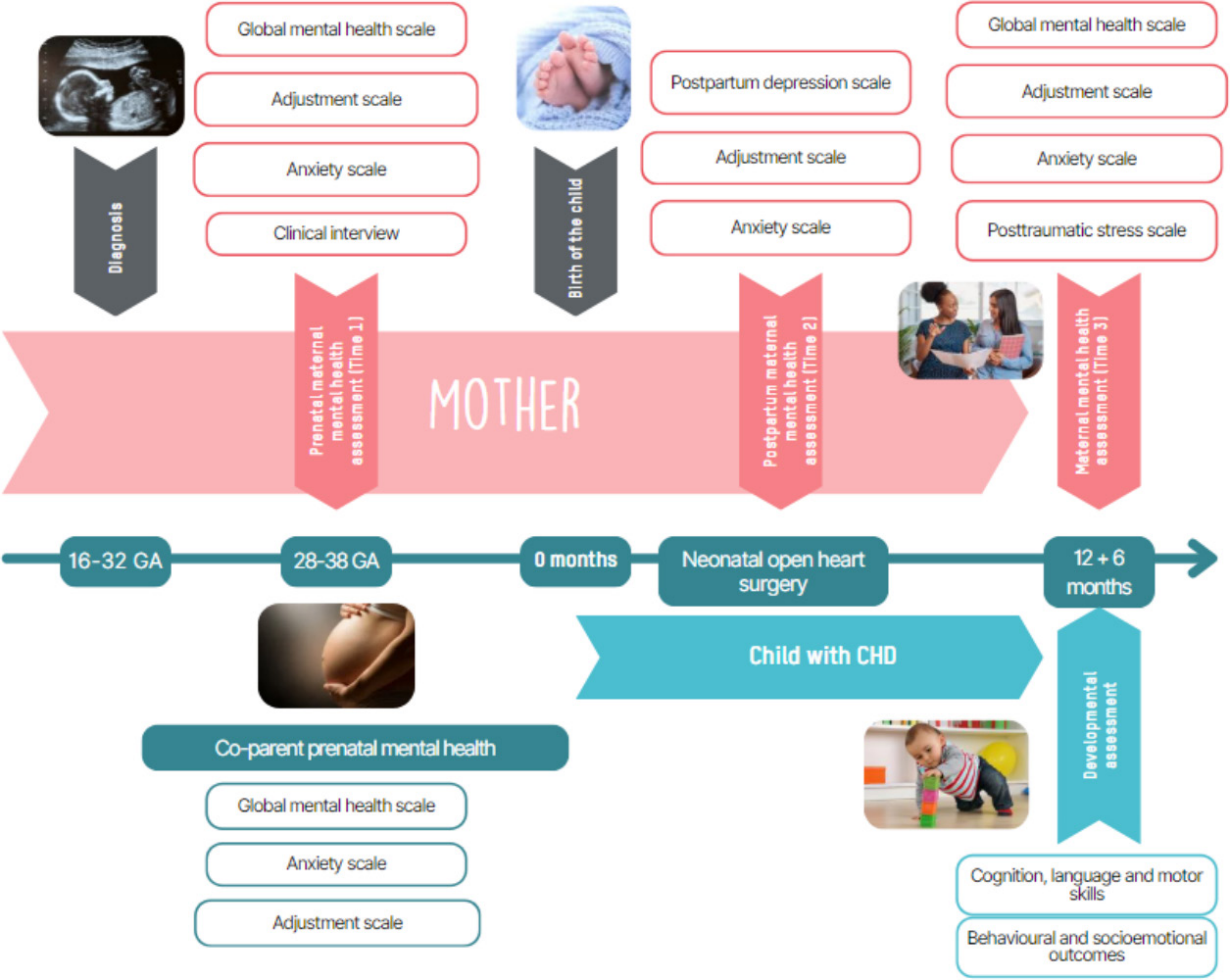
Study visit and data collection schedule. CHD, congenital heart disease; GA, gestational age.

Figure 1 indicates the study visit collection. At Time 1 (third trimester of pregnancy) after obtaining agreement to participate and signing written consent, a semi-structured psychological interview, lasting about 45 min will be conducted. Three main themes will be investigated: (1) diagnosis of CHD, experience of pregnancy and mental health (ie, post-diagnosis psychological experience, existing family resources); (2) perspectives and projections (ie, how to imagine one’s life after one’s child’s heart surgery) and (3) expectancies towards a maternal mental health intervention in the context of prenatal CHD diagnosis. The prenatal psychological interviews may be conducted on site at each centre before or after a follow-up clinical consultation, or by telehealth in order to facilitate the participation of expectant mothers in the study. Self-report questionnaires assessing psychological state (SCL-90-R), anxiety (STAI-Y) and coping strategies (CISS), as well as an information questionnaire (sociodemographic aspects and lifestyle) will be collected. This information questionnaire will collect information about level of education, employment status, marital status, family situation (living alone or with others, number of children), geographical area of residence and distance between home and maternity hospital, available resources (type of support that can be requested and obtained, whether practical, financial or emotional), lifestyle habits, including smoking, alcohol and drugs, physical activity and sleep quality. The questionnaires take approximately 30 min to complete. The time required to complete all the questionnaires is estimated at around 30 min for the mother and 30 min for the father or co-parent. The total duration of the visit is estimated at between 45 min and 1 hour 15 min for the mothers and 30 min for the co-parent. Participants have the possibility to complete the questionnaires at home, after receiving the instructions.

At Time 2 (after surgery, before the child is discharged from hospital), a post-partum depression questionnaire (EPDS), an anxiety questionnaire (STAI-Y) and a coping scale (CISS) will be administered to the participants, at the most convenient time for them. Finally, an information note concerning the child’s next visit will be given to the parents along with parental consent for the child’s participation.

**Figure 2:**
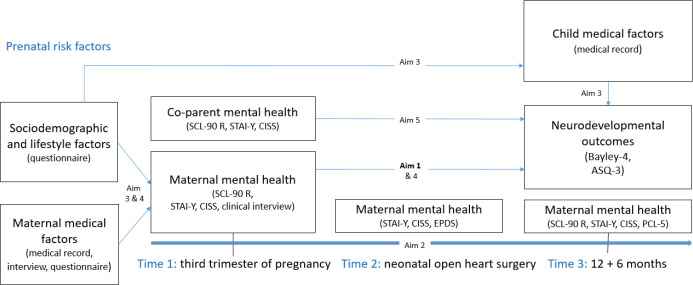
Diagram of variables of interest and their measurement. ASQ-3, Ages and Stages Questionnaire-Third Edition; Bayley-4, Bayley Scales of Infant and Young Child Development, 4th edition; CISS, Coping Inventory for Stressful Situations; EPDS, Edinburgh Postnatal Depression Scale; PCL-5, Posttraumatic Stress Disorder Checklist for DSM-5; SCL-90 R, Symptom Check-List-90 R; STAI-Y, State-Trait Anxiety Inventory.

At Time 3, a follow-up consultation will be conducted with mothers and their 12 to 18 months old child. The mother will be offered a psychological state questionnaire (SCL-90-R), an anxiety scale (STAI-Y), a coping scale (CISS) and a post-traumatic stress scale (PCL-5). A neurodevelopmental examination using the standardised ASQ-3 and Bayley-4 tests will be conducted, including an assessment of the child’s behavioural and socio-emotional development, and their neurodevelopment (cognition, language, fine and gross motor skills, as well as psychosocial development). No measures will be collected for the co-parent at Time 3. Figure 2 summarises the measurements.

A referral psychiatrist will be appointed in the associated centres to respond to urgent psychiatric needs (ie, severe pathological levels of anxiety and/or depressive disorders identified on the questionnaires and/or any other situation requiring immediate or urgent clinical care).

To enhance participant inclusion, we rely on the involvement of the medical team who conducts prenatal diagnoses with dedicated consultations. We also benefit from the close involvement of patient organisations such as Petit Cœur de Beurre Association. They support clinical research by communicating with families about our study. Clinical research assistants regularly contact families, which allows for close monitoring of children during cardiology consultations. To make it easier for them to attend appointments, travel expenses can be covered by the family association, thanks to a dedicated budget allocated to the association to help families of children with CHD. In addition, neurocardiac centres already have a joint organisation with cardiological follow-up, which will facilitate patient visits.

### Statistical Plan

#### Sample size and power considerations

Our main objective is to determine the association between the antenatal maternal psychological state score measured by the SCL-90-R and neurodevelopmental outcomes at age 1 measured via the Bayley-4 and the neurodevelopmental state score of children with CHD at age 1. We hypothesise a Pearson correlation of r=0.45 (or R2=0.452= 0.202 for linear regression) between the maternal mental health score and the global cognitive score of children with CHD at age 1. The inclusion of 10 covariates in the multiple linear regression analysis with an increase in R2 of 0.20 requires the inclusion of a total of 72 dyads for a power of 90% and an alpha risk of 5% (NQuery software, ROT4-1). As an attrition rate of 20% is expected (due to different causes such as death of the child, or mother or child drop out), a total of 87 dyads will be included.

### Data analysis plan

#### Main objective

The relationship between the score on the SCL-90-R prenatal global mental health self-report questionnaire, taking into account the other factors likely to influence the neurodevelopment score (sociodemographic, medical and psychological factors), and the child’s neurodevelopment score at age 1 on the standardised Bayley-4 test, will be assessed using multiple linear regression after checking the conditions of application. The main explanatory variable will be the SCL-90-R global score and the variable to be explained will be the child’s neurodevelopment score. The multivariate results will be reported using Beta coefficients and LSMEANS (adjusted means) and their 95% CI. The analyses will be conducted using SAS 9.4 and R 4.5.

#### Secondary objectives

##### Aims 2 and 3

In order to study changes over time (ie, evolution of maternal mental health) in the scores obtained on the self-report questionnaires (SCL-90-R, STAI-Y, CISS, PCL-5, EPDS) completed by the mother and to study changes as a function of medical, sociodemographic and lifestyle factors, a linear mixed model will be applied. This model takes into account the random effect induced by the analysis of three repeated measurements. The cofactors tested (fixed effects) will be the variables likely to influence the scores on these self-report questionnaires (medical, sociodemographic and lifestyle). The results of the linear mixed model are expressed for each factor in the form of a coefficient corresponding to the variation brought about by this factor on the basal score (intercept). A positive coefficient indicates a score-raising effect, while a negative coefficient indicates a score-lowering effect. For the time effect, the coefficient reflects the variation during the follow-up, while for the other factors, the estimate reflects the variation induced by the presence of the factor. The interactions between the co-factors and time will also be tested. A similar methodology will be applied for each score.

##### Aim 4

We will use a structural equation model to estimate simultaneously the relationships among maternal mental health (as captured by SCL-90-R, STAI-Y, CISS, PCL-5 and EPDS scores), prenatal factors (medical, sociodemographic and psychological) and child development (Bayley-4 and ASQ-3).

The structural pathways linking these three latent constructs, whereby maternal mental health impacts prenatal factors, which in turn influence the child’s development, while also allowing for a residual direct effect of maternal mental health on developmental outcomes.

We will quantify the mediating role of each block of prenatal factors by estimating the proportion of the effect of maternal mental health on child development that is transmitted through those factors. The model will be fitted using robust maximum likelihood estimation, with bootstrapping to derive CIs for indirect effects.

##### Aim 5

The GSI of the SCL-90-R will be used as an indicator of co-parent mental health. It is a standardised score with a T-score (mean 50, SD 10). The Bayley-4 cognitive score will be used as an indicator of neurodevelopment in children with CHD at age 1. This is an exploratory analysis, independent of the primary outcome.

## DISCUSSION

This article presents the background and design of the first multicentre longitudinal study investigating the impact of prenatal maternal mental health on 1-year neurodevelopmental outcomes in children with CHD who undergo neonatal open-heart surgery (ie, children at higher neurodevelopmental risk). The risk of deterioration in maternal mental health is particularly high in the case of a complex cyanotic CHD diagnosis in the infant. We will evaluate maternal mental health at three time points, and its effect on the neurodevelopment in the child with CHD.

Our study is the first research initiative to investigate the link between prenatal parental mental health and neurodevelopmental outcomes in children with complex cyanotic CHD at age 1. Understanding the risk factors for neurodevelopmental impairments, from the prenatal period onwards, is a public health priority. A multifaceted approach including psychological modifiable factors is needed to improve these impairments. The psychological data collected in this study are necessary for better anticipating preventive strategies for this high-risk population, particularly by enhancing our knowledge about the critical effects of prenatal maternal stress on children’s neurodevelopment. It thus will enable us to better target preventive strategies for the psychological well-being of families, involving adapted prevention protocols in fetal and congenital cardiology units for parents whose child is diagnosed with complex cyanotic CHD. We anticipate that long-term follow-up of this cohort is needed to better characterise the potential long-lasting effects of prenatal parental stress on neuropsychological development in children with CHD. Ultimately, our overarching goal is to propose interventions to improve maternal mental health, as a potentially modifiable protective factor that can mitigate or prevent neurodevelopmental impairments in youth with CHD.

### Ethics and dissemination

This study is sponsored by the French National Institute of Health and Medical Research. It was granted approval by local Ethics Committee ‘Comité de Protection des Personnes Ouest IV’ (file number 2024-A02138-39) on 5 November 2024 and is registered in a public trials registry (NCT06711666). The Neuro-Moms CHD project targets a critical public health question regarding maternal mental healthcare for children with chronic diseases. Heart disease being the first cause of congenital anomalies, the societal implications are expected to be broadly communicated not only within the scientific community but also the lay public. After final analysis, the results will be published in the form of scientific articles in peer-reviewed journals and presented at national and international conferences. Any publication or communication (oral or written) will be decided by mutual agreement between the co-ordinating and PIs, the scientific managers and the sponsor, and will comply with the international recommendations: ‘Recommendations for the Conduct, Reporting, Editing, and Publication of Scholarly work in Medical Journals’ (http://www.icmje.org/recommendations). All parents/guardians and children will give written informed consent or assent to participate prior to entering the study.

### Study progress

The study will start in June 2025. This is Protocol V.1.0, 24 September 2024. Potential protocol amendments will be communicated and updated in clinicaltrials. gov. https://clinicaltrials.gov/study/NCT06711666?id=NCT06711666&rank=1.
