# Supplementary material for: Prenatal maternal mental health and neurodevelopment in congenital heart disease in France: the neuro-moms CHD multicentre prospective study protocol
Source: BMJ Open. 2025 Dec 23;15(12):e104889. doi: 10.1136/bmjopen-2025-104889 (PMC12730764; doi:10.1136/bmjopen-2025-104889)
Supplement: Supplementary file 1 [file bmjopen-2025-104889.reviewer_comments.pdf]

PEER REVIEW HISTORY

BMJ Open publishes all reviews undertaken for accepted manuscripts. Reviewers are asked to complete a checklist review form and are provided with free text boxes to elaborate on their assessment. These free text comments are reproduced below.

ARTICLE DETAILS

Title (Provisional)

Prenatal Maternal Mental Health and Neurodevelopment in Congenital Heart Disease in France: The Neuro-Moms CHD Multicentre Prospective Study Protocol

Authors

Deninotti, Julie; Derridj, Neil; Martins, Sylvie; Laux, Daniela; Stos, Bertrand; Levy, Marilyne; Desnous, Beatrice; Guillaumont, Sophie; Amédro, Pascal; Chabaneix, Julie; Pfister, Marion; Marguin, Gaelle; Desmure, Gwladys; Vincenti, Marie; Bonnet, Damien; Calderon , Johanna

---

VERSION 1 - REVIEW

**Reviewer**	**1**
**Name**	**Shikata, Fumiaki**
**Affiliation**	**The University of Tokyo, Cardiac Surgery**
**Date**	**01-Sep-2025**
**COI**	**None**

The authors rightly note that neurodevelopment is a long-term trajectory. While the one-year follow-up is an excellent starting point, I would strongly recommend expanding the study to include follow-up assessments at older ages, such as preschool and school age. This would provide more robust and long-lasting insights into the impact of prenatal stress on long-term neurodevelopmental outcomes in children with CHD.

**Reviewer**	**2**
**Name**	**Williams, Tricia**
**Affiliation**	**The Hospital for Sick Children**
**Date**	**05-Nov-2025**
**COI**	**None**

Thank you for inviting me to review the manuscript, Prenatal Maternal Mental Health and Neurodevelopment in Congenital Heart Disease: The Neuro-Moms CHD Multicenter

Prospective Study Protocol. Overall, this protocol is exceptionally well written, with a compelling rationale, robust methodology, and the potential to significantly inform neurodevelopmental care before birth, after birth, and during early childhood for high-risk CHD populations. The findings are poised to greatly advance our understanding of the factors influencing parental mental health throughout the perinatal period, its trajectory after birth, and how it affects early neurodevelopmental outcomes in children with CHD.

I offer several general comments for the authors' consideration below. Broadly, however, the protocol provides limited discussion on how social determinants affect maternal mental health psychosocial distress and children's neurodevelopmental outcomes. While these factors appear in Figure 2, they are not clearly detailed in the objectives or hypotheses. For thorough risk stratification and targeted intervention across these trajectories, accounting for social determinants is crucial. Could deprivation indices from census data, which extend beyond self-reported demographic information, be incorporated—perhaps on an exploratory basis?

Once again, thank you for inviting me to review this work. I wish the authors every success in their research.

I comment on each section respectively below.

Introduction

The introduction is beautifully written with strong and selective citations and a clear rationale to support the proposed study protocol.

Aims & Methods

Aim 1 is however I only recommend that the authors may wish to increase specificity in their hypothesis and whether they think that maternal mental health may have differential impact across the Bailey measures (cognitive, language, motor)?

Similarly in aim 2, are the authors proposing that there will be general reductions across the different sub scales of their measure of maternal mental health? In the introduction and discussion, the authors review the substantial number of individuals that suffer from post-partum depression and deterioration, so their hypothesis of improvement seem counter to this.

Is the measure of coping to general life stress or are there any specific questions related to coping with significant medical diagnosis?

Is language considered as an inclusion or exclusion criterion, and will interpreter services be provided to support participation?

How will multi-site coordination be implemented?

What are the investigators mitigation strategies for this trial to optimize inclusion and minimize attrition?

Analyses: Excellent.

Discussion

How can using the symptom checklist global distress as a primary outcome inform preventative interventions if it only summarizes general distress without distinguishing anxiety, depression, or other psychopathologies?

I'm also interested in how this study plans to adjust for pre-pregnancy mental health history. Will it also capture whether participants have access to any psychosocial support?

VERSION 1 - AUTHOR RESPONSE

Reviewer: 1
Dr. Fumiaki Shikata, The University of Tokyo
Comments to the Author:
The authors rightly note that neurodevelopment is a long-term trajectory. While the one-year follow-up is an excellent starting point, I would strongly recommend expanding the study to include follow-up assessments at older ages, such as preschool and school age. This would provide more robust and long-lasting insights into the impact of prenatal stress on long-term neurodevelopmental outcomes in children with CHD.

Response: *Thank you very much for this thoughtful comment. Indeed, even though our current funding targets a 12–18-month follow-up, we had added the need to re-evaluate children at later stages of development in our strengths and limitation section. We have also emphasized the need for long term follow-up in our discussion.*

Reviewer: 2
Dr. Tricia Williams, The Hospital for Sick Children
Comments to the Author:
Thank you for inviting me to review the manuscript, Prenatal Maternal Mental Health and Neurodevelopment in Congenital Heart Disease: The Neuro-Moms CHD Multicenter Prospective Study Protocol. Overall, this protocol is exceptionally well written, with a compelling rationale, robust methodology, and the potential to significantly inform neurodevelopmental care before birth, after birth, and during early childhood for high-risk CHD populations. The findings are poised to greatly advance our understanding of the factors influencing parental mental health throughout the perinatal period, its trajectory after birth, and how it affects early neurodevelopmental outcomes in children with CHD.
I offer several general comments for the authors' consideration below.

Broadly, however, the protocol provides limited discussion on how social determinants affect maternal mental health psychosocial distress and children's neurodevelopmental outcomes. While these factors appear in Figure 2, they are not clearly detailed in the objectives or hypotheses. For thorough risk stratification and targeted intervention across these trajectories, accounting for social determinants is crucial. Could deprivation indices from census data, which extend beyond self-reported demographic information, be incorporated—perhaps on an exploratory basis?

Response: *Thank you for your thoughtful comments and review of our paper. Indeed, social determinants may play a crucial role in the relationship between parental mental health and*

neurodevelopment in children at high-risk for neurocognitive impairment, such children with CHD. In our study, this information is collected via the sociodemographic questionnaires and include data regarding level of education, employment status, marital status, family situation (living alone or with others, number of children), geographical area of residence and distance between home and maternity hospital, available resources (type of support that can be requested and obtained, whether practical, financial, or emotional), but also lifestyle habits, including smoking, alcohol, and drugs, physical activity, and sleep quality. We will include social determinants in our analyses, as potential co-variates (pages 14-15 of our manuscript). Deprivation or vulnerability indices from census national data will be integrated on an exploratory basis, following the references from the French National Institute of Statistics and Economic Studies (Insee). We have added this to the Covariate measure section (pages 14-15).

Once again, thank you for inviting me to review this work. I wish the authors every success in their research.
I comment on each section respectively below.
Introduction
The introduction is beautifully written with strong and selective citations and a clear rationale to support the proposed study protocol.
Aims & Methods

Aim 1 is however I only recommend that the authors may wish to increase specificity in their hypothesis and whether they think that maternal mental health may have differential impact across the Bailey measures (cognitive, language, motor)?

Our primary outcome is the Bayley cognitive score as it is our primary hypothesis that overall cognitive development may be predominantly affected by prenatal psychological distress. However, analyses will also be conducted on other Bayley-IV measures (language and motor). By doing so, we will consider a potential differential impact across these measures.
Similarly in aim 2, are the authors proposing that there will be general reductions across the different sub scales of their measure of maternal mental health? In the introduction and discussion, the authors review the substantial number of individuals that suffer from post-partum depression and deterioration, so their hypothesis of improvement seem counter to this.

Response: *Thank you for this question. We clarified our hypothesis that psychological distress will remain high, per questionnaire standards, at all-time points (prenatal, post-surgical, and 12-months post-partum). However, we do hypothesize that symptoms of psychological distress will tend to decrease over this period. Indeed, the prenatal diagnosis of CHD has been identified as a critically high period of stress in these pregnancies. The tendency after the heart operation (once the child is considered stable and "repaired") is to have a relative decrease in acute anxiety. We will test this hypothesis for this sample in Neuro-Moms CHD. We added a clarification for this in our hypothesis on Page 8: "We predict that maternal psychological distress will decrease over time, as we expect that the most acute period of stress will be closest to the initial diagnosis of fetal CHD (T1, T2 and T3)."*

Is the measure of coping to general life stress or are there any specific questions related to coping with significant medical diagnosis?

Response: *This is a coping measure that applies to life stress in general, as it reflects participants' usual coping style and the extent to which they will demonstrate resilience in general. Aspects relating to more specific coping with a prenatal CHD diagnosis are addressed during the semi directive interview.*

Is language considered as an inclusion or exclusion criterion, and will interpreter services be provided to support participation?
Response: *The inability to understand all the questionnaires due to severe language barriers is unfortunately, an exclusion criterion, as questionnaires are not necessarily standardized, and all evaluators do not speak languages other than French/English.*

How will multi-site coordination be implemented?
What are the investigators mitigation strategies for this trial to optimize inclusion and minimize attrition?
Response: *Multi-site coordination in ensured by the French National Institute of Health and Medical Research, Inserm by Dr. Calderon. Each hospital site will have a designated local PI that will ensure local regulatory compliance.*
To enhance participant inclusion, we rely on the involvement of the medical team that conducts prenatal diagnoses with dedicated consultations, as well as psychological consultations in Bordeaux, which are currently being set up at Necker Hospital. We also benefit from the close involvement of patient organizations such as Petit Cœur de Beurre Association. They support clinical research by communicating with families about our study. Clinical research assistants regularly contact families, which allows for close monitoring of children during cardiology consultations. To make it easier for them to attend appointments, travel expenses can be covered by the family association, thanks to a dedicated budget allocated to the association to help families of children with CHD. In addition, neurocardiac centers already have a joint organization with cardiological follow-up, which will facilitate patient visits.

Analyses: Excellent.
Discussion
How can using the symptom checklist global distress as a primary outcome inform preventative interventions if it only summarizes general distress without distinguishing anxiety, depression, or other psychopathologies? I'm also interested in how this study plans to adjust for pre-pregnancy mental health history. Will it also capture whether participants have access to any psychosocial support?

Response: *We thank this reviewer for the opportunity to clarify. The overall score is the outcome we use in relation to the child's subsequent neurodevelopment. This checklist includes several areas that we will also analyze individually to better target prenatal interventions, and it is supplemented by other more specific questionnaires, such as the STAI-Y for anxiety, the EPDS for postnatal depression and the PCL-5 for posttraumatic stress. In addition, data from the semi-structured interview will also be informative in refining the existing symptoms in our participants. Data on psychological/psychiatric history prior to pregnancy is collected through the information questionnaire, as well as any consultation with a mental health professional.*
